# Laboratory Risk Indicator for Necrotizing Fasciitis of the Oro-Cervical Region (LRINEC-OC): A Possible Diagnostic Tool for Emergencies of the Oro-Cervical Region

**DOI:** 10.1155/2019/1573453

**Published:** 2019-11-14

**Authors:** Masaru Ogawa, Satoshi Yokoo, Yu Takayama, Jun Kurihara, Takaya Makiguchi, Takahiro Shimizu

**Affiliations:** Department of Oral and Maxillofacial Surgery and Plastic Surgery, Gunma University Graduate School of Medicine, 3-39-22 Showa-machi, Maebashi, Gunma 371-8511, Japan

## Abstract

**Aim:**

Oro-cervical necrotizing fasciitis (OCNF) treatment requires early surgical debridement and opening of the wound, and therefore, early diagnosis is very important. The Laboratory Risk Indicator for Necrotizing Fasciitis (LRINEC) score based on blood test data has recently been proposed as an auxiliary diagnostic tool. However, in some cases, it is difficult to diagnose OCNF. We performed a pooled analysis of patients with OCNF at Gunma University Hospital and literature cases, with the goal of designing a new auxiliary diagnostic tool for OCNF by adding physical characteristics of the oro-cervical region to blood test data in the first examination.

**Methods:**

Univariate and multivariate logistic regression was used to select predictors of OCNF. The LRINEC-Oro-Cervical (OC) score was then designed using correlation coefficients of items selected in logistic regression analysis. A cutoff value for the LRINEC-OC score was determined using receiver operating characteristic (ROC) curve analysis.

**Results:**

CRP, WBC, Cr, and skin flare in the cervical and precordial regions were extracted as independent factors (*p* < 0.05) and evaluated as predictors of OCNF. The LRINEC-OC score for the prediction of OCNF was designed using the regression coefficients in logistic analysis. The cutoff value for the LRINEC-OC score was 6 points with a sensitivity of 88.5% and a specificity of 93.4%, and the AUC was 0.909.

**Conclusion:**

Delays in diagnosis and surgical treatment for OCNF led to a fatal prognosis, and the potential utility of the LRINEC-OC score for improving the prognosis was shown in this study.

## 1. Introduction

Oro-cervical necrotizing fasciitis (OCNF) is a soft-tissue infection of the oro-cervical region by an oral indigenous anaerobe that causes extensive necrosis of the fascia and subcutaneous tissue [1–3]. Histopathological examination is required for definite diagnosis of necrotizing fasciitis. OCNF is almost always accompanied by gas production and readily spreads to the mediastinum and great vessels due to the anatomical characteristics of the cervical region and becomes fatal. Treatment requires early surgical debridement and opening of the wound, and therefore, early diagnosis is very important. The prognosis is likely to be poor if surgery is not performed within 24 hours after onset [4]. However, in some cases, it is difficult to differentiate the early stage of OCNF from cellulitis. Confirmation of gas production by CT is useful for differential diagnosis of these diseases, but this is not a simple examination and preliminary screening for OCNF is needed.

The Laboratory Risk Indicator for Necrotizing Fasciitis (LRINEC) score based on blood test data has recently been proposed as an auxiliary diagnostic tool [5–8]. However, Thomas and Meyer [9] found that the LRINEC score is unreliable for the head and neck region. Therefore, we performed a pooled analysis of patients with OCNF at the Department of Oral and Maxillofacial Surgery, Gunma University Hospital, and cases from the recent literature to establish the clinical and serological characteristics of OCNF in emergency cases.

The purpose of this study is to design a new auxiliary diagnostic tool for OCNF by adding physical characteristics of the oro-cervical region to blood test data in the first examination for improving the prognosis of OCNF.

## 2. Materials and Methods

The OCNF group (*n* = 26) included 7 patients treated at the Department of Oral and Maxillofacial Surgery, Gunma University Hospital, from January 2010 to December 2017 and 19 Japanese patients described in the previous reports [10–27]. These cases were identified in a literature search carried out using “necrotizing fasciitis,” “gas gangrene,” “gas-producing cellulitis,” “head and neck region,” and “odontogenic” as key words in PubMed and the Japan Medical Abstracts Society (JMAS) Web. The non-OCNF group (*n* = 121) consisted of patients with severe cellulitis who were treated with intravenous antibiotics for ≥72 hours in the same period of OCNF treatment. Cases with mild cellulitis resolved by oral antibiotics were not included in the study.

Age, gender, blood test data, and physical findings in the emergency examination were investigated in the OCNF and non-OCNF groups. Blood tests were performed for C-reactive protein (CRP), white blood cell count (WBC), hemoglobin (Hb), serum creatinine (Cr), liver enzymes (GOP and GOT), blood glucose (Glu), and serum protein (Alb). Physical findings included body temperature, position of the causative tooth, and presence or absence and range of skin flare from the cheek to the precordial region. All data were obtained retrospectively from medical records at Gunma University Hospital and previous reports.

For comparison between the OCNF and non-OCNF groups, the above items were first subjected to univariate analysis using a cross table with *χ*^2^ test for independence. Binary data as explanatory variables were prepared based on the criteria for the original LRINEC score. Physical findings were analyzed as positive (+) or negative (−). Next, the items with significant differences between the two groups were analyzed using logistic regression analysis with the forward selection method. Multicollinearity was judged to be present if the absolute correlation coefficient between two explanatory variables was <0.7. A variance inflation factor (VIF) was calculated to eliminate multicollinearity between explanatory variables. Explanatory variables with VIF <10, which indicates no multicollinearity, were used to ensure that no variables had multicollinearity. The LRINEC-Oro-Cervical (OC) score was then designed using correlation coefficients of the items selected in logistic regression analysis. A cutoff value for the LRINEC-OC score was determined using receiver operating characteristic (ROC) curve analysis. Statistical analysis was performed with SPSS for Windows, ver.25 (IBM Corp., Armonk, NY). *p* < 0.05 was considered to denote significance.

## 3. Results

### 3.1. Patient Background

The patient backgrounds in the OCNF and non-OCNF groups are shown in Table 1. Age, gender, thrombocytopenia, and the presence of malignant disease did not differ significantly between the groups. Seven patients with OCNF treated at our hospital survived, but 3 patients with OCNF in previous reports died, giving a mortality rate of 11.5%, which was significantly higher in the OCNF group (*p*=0.006).

### 3.2. Analysis of Predictors of OCNF

Items based on the criteria for the original LRINEC score and physical findings were subjected to univariate analysis. CRP, WBC, Cr, Glu, Alb, and skin flare region (cheek, cervical, and precordial) showed significant differences between the OCNF and non-OCNF cases (*p* < 0.05) (Table 2). There was no multicollinearity among these 8 items, and therefore, logistic regression analysis with the forward selection method was carried out with all 8 items as explanatory variables. CRP, WBC, Cr, and skin flare in the cervical and precordial regions were extracted as independent factors (*p* < 0.05) and evaluated as predictors of OCNF. The multivariate-adjusted odds ratios were high for CRP (34.13, 95% confidence interval (CI) 5.67–205.52) and Cr (34.30, 95% CI 2.83–416.17), indicating that these variables were the most important predictive factors (Table 3).

### 3.3. Application of the LRINEC-OC Score

**Figure 1:**
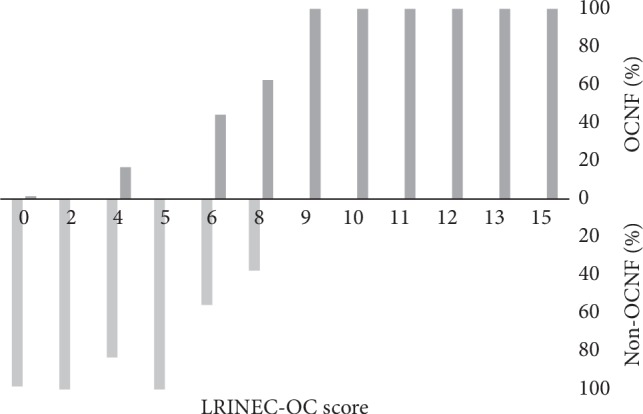
Distribution of LRINEC-OC scores in patients with and without OCNF. The percentages of OCNF patients with scores of 9–15 were 100%.

**Figure 2:**
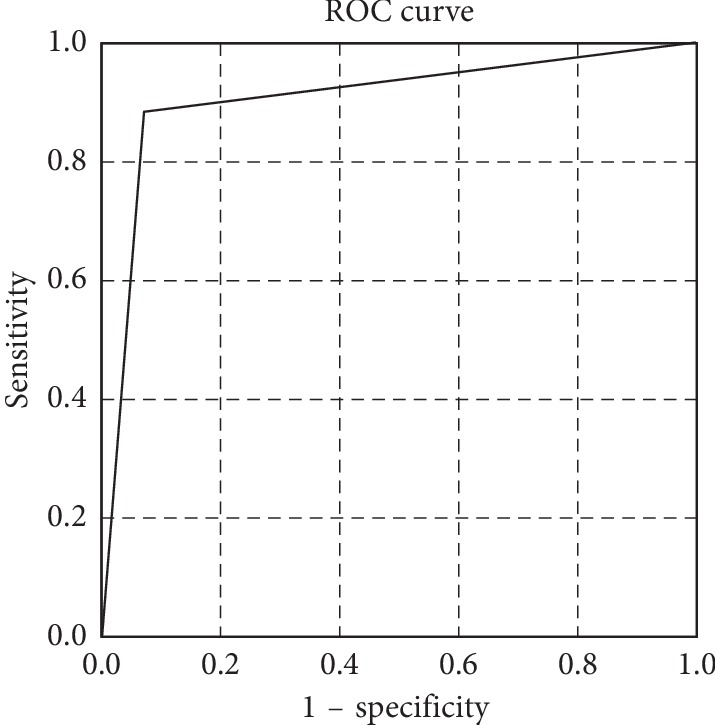
Receiver operating characteristic (ROC) curve based on a cutoff LRINEC-OC score of ≥6 in predicting the presence of necrotizing fasciitis. Area under the curve for our model is 0.909.

The LRINEC-OC score for prediction of OCNF was designed using the regression coefficients in logistic analysis. The maximum total score was 15 (Table 4). The distributions of LRINEC-OC scores in the OCNF and non-OCNF groups are shown in Figure 1. Because all OCNF patients had scores of 9–15, a score of 9–15 was defined as a high risk for OCNF. The cutoff value for moderate risk was determined using receiver operating characteristic (ROC) curve analysis. In this analysis, the sensitivity and specificity at a LRINEC-OC cutoff of 6 were 88.5 and 93.4%, respectively, and the AUC was 0.909 (Figure 2). Therefore, scores of ≤5, 6–8, and 9–15 were defined as low-, moderate-, and high-risk based on these data (Table 4).

## 4. Case Presentation

**Figure 3:**
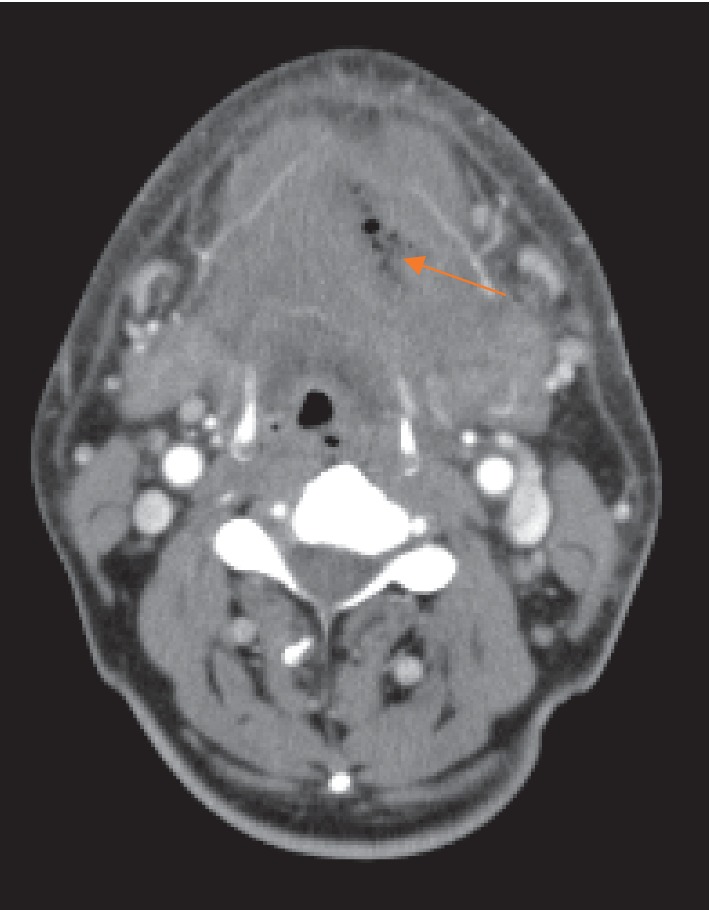
Typical CT image in the first examination. Gas was noted in the left submandibular region over the submental region.

**Figure 4:**
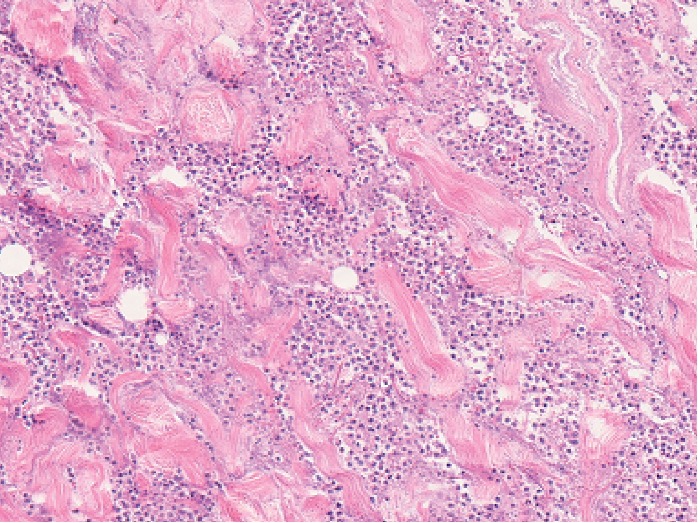
Histopathological findings. Histopathology of debrided tissues showing massive infiltration of neutrophils in muscular layers with necrosis (hematoxylin and eosin stain; magnification ×20).

**Figure 5:**
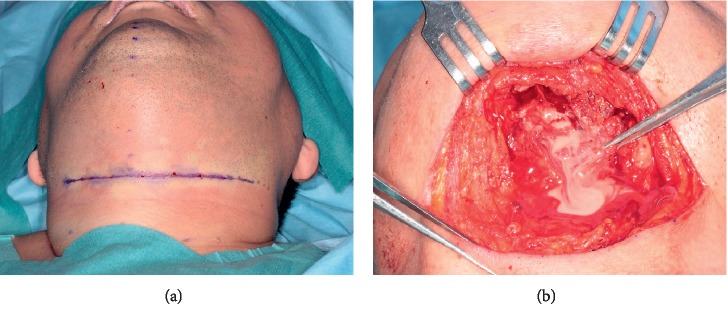
Intraoperative findings. The wound was opened under general anesthesia. Extensive colliquative necrosis and malodorous drainage were noted in muscle tissue. Debridement of the necrotic tissue was carried out, and the wound was left open.

A 61-year-old Japanese male with swelling and pain in the left mandibular region consulted with the Department of Oral and Maxillofacial Surgery, Gunma University Hospital. His Glasgow Coma Score was 15 at this visit, indicating no disturbance of consciousness. On physical examination, skin flare and diffuse swelling accompanied by tenderness were noted in the left lower face over the cervical region. However, the swelling was not severe and no precordial skin flare was noted. Maximum opening of the mouth was 24 mm, and swallowing pain was noted. In the oral cavity, swelling and mucosal flare were noted around the left lower third molar. The case could not be diagnosed as OCNF or non-OCNF based on the local findings. Blood tests gave a WBC count of 15,000/*μ*L; CRP 31.5 mg/dL; and Cr 0.87 mg/dL. The LRINEC-OC score was 8, suggesting a moderate risk of OCNF (the original LRINEC score was 5, indicating a low risk). The patient was admitted on the same day, and CT was carried out. Gas was noted in the left submandibular region over the submental region (Figure 3), based on which OCNF was clinically diagnosed. On the same day, the wound was opened under general anesthesia. Extensive colliquative necrosis and malodorous drainage were noted in muscle tissue. The histopathological diagnosis was NF (Figure 4). Debridement of the necrotic tissue was carried out, and the wound was left open (Figure 5). Wound bed preparation was continued under hospitalization, and the patient was discharged on the 65th hospital day.

## 5. Discussion

Necrotizing fasciitis (NF) is a rare, severe, skin, soft-tissue infection, in which extensive necrosis of the superficial fascia and subcutaneous tissue progresses rapidly [1]. Histopathological examination is essential to diagnose with NF distinctly. NF is almost accompanied by gas production. NF develops concomitantly with ischemia and infection and almost always in the extremities and thoracoabdominal region. In the oro-cervical region, NF develops less likely due to the abundant blood supply [28]. The fascia of the head and neck region are comprised of areolar tissue, which is separated by the superficial cervical fascia and deep cervical fascia. The deep cervical fascia is classified into a superficial layer (investing fascia), a middle layer (buccopharyngeal fascia), and a deep layer (prevertebral fascia). The compartment surrounded by the deep cervical fascia is called the space of the head and neck. Because the retropharyngeal space or danger space of the head and neck is topologically continuous with the mediastinum, infection of this space is highly likely to cause fatal complications such as mediastinitis. Infection of the danger space sometimes extends to not only the mediastinum and pericardium but also the retroperitoneum. Death occurs in 8–15% of OCNF cases due to infection of the mediastinum [29–31]. Therefore, the prognosis of OCNF depends on early diagnosis and appropriate emergency treatment, i.e., prevention of the spread of infection to the mediastinum by surgical debridement. Liu et al. [32] showed the importance of surgical debridement within 24 hours based on mortality in such cases of 26%, compared to 45.9% in those treated after 24 hours.

Differentiation of OCNF from general cellulitis is difficult in the early stage [33]. A finding of gas on CT is useful for diagnosis, but there is hesitation regarding performance of CT in the early stage in many clinical cases. Therefore, there is a need for screening of patients with suspected OCNF based on an emergency examination, with subsequent appropriate emergency treatment. Wong et al. [5–7] and Liao et al. [8] recently reported the LRINEC score as an auxiliary diagnostic tool for early differentiation of NF and non-NF cases. In the only evaluation of this score for cases (17 NF and 70 non-NF) in the oro-cervical region, Thomas and Meyer [9] found that a cutoff of 6 for the LRINEC score had sensitivity, specificity, positive predictive value (PPV), and negative predictive value (NPV) of 56%, 60%, 25%, and 85%, respectively, for diagnosis of NF, and concluded that the score was not useful. In our patients, the original LRINEC score was ≤5 in 3 of the 7 patients diagnosed with OCNF, again suggesting that this score is limited for diagnosis. This may be because Wong et al. [5] examined NF in the extremities and trunk, but not in the oro-cervical region. Abdullah et al. [34] reviewed 18 papers with the aim of evaluating the reliability of the LRINEC score, but this study did not focus on the oro-cervical region. The type of pathogenic bacteria and pattern of spread of infection differ between these sites, and NF and OCNF may have a different pathogenesis.

With this background, we developed a new NF diagnostic tool that is specific for the oro-cervical region by adding characteristic physical findings to blood test data. Statistical analysis showed that CRP, Cr, WBC, and skin flares of the cervical and precordial regions were independent predictors of OCNF, and the odds ratios for CRP and Cr were particularly high, suggesting that these are the most important factors. A high CRP level also occurs in general cellulitis, but the mean CRP level of 28.1 mg/dL in the OCNF cases was extremely high, which reflects the severe infection and tissue destruction in OCNF. A high Cr level suggests that the risk of renal disorder is high in patients with OCNF.

Liao et al. [8] retrospectively investigated 233 patients with NF and 1,394 patients with severe cellulitis treated in the same period under conditions similar to those in Wong et al. [5]. The sensitivity, specificity, PPV, and NPV were 59%, 84%, 38%, and 93%, respectively, for a LRINEC score ≥6. In this study, the sensitivity, specificity, and AUC were 88.5%, 93.4%, and 0.909, respectively, for a LRINEC-OC score ≥6, indicating that the LRINEC-OC score is more effective than the original LRINEC score for screening of OCNF in an emergency examination. In moderate- to high-risk patients with a LRINEC-OC score ≥6, CT should be immediately carried out to examine the presence of gas since this is useful for the diagnosis of OCNF. Gas was confirmed on the first CT examination in all 7 of our patients, and this contributed to surgical treatment being performed within 24 hours and ultimately resulted in survival.

As a limitation of the study, this is a retrospective pooled analysis of cases from recent literature and our own cases. A prospective study is required to validate the usability of the LRINEC-OC score.

## 6. Conclusions

The LRINEC-OC was designed as a laboratory risk indicator to screen for necrotizing fasciitis of the oro-cervical region by adding characteristic physical findings for OCNF to blood test data. Delays in diagnosis and surgical treatment for OCNF lead to a fatal prognosis, and the potential utility of the LRINEC-OC score for improving the prognosis was shown in this study.

## Figures and Tables

**Table 1:** Patient background in the OCNF and non-OCNF groups.

Variable	OCNF (*n* = 26)	Non-OCNF (*n* = 121)	*p* value
Age (years)	61.9 (22–88)	50 (8–82)	0.173
Gender			0.844
Male (%)	13 (50.0)	57 (47.1)	
Female (%)	13 (50.0)	64 (52.9)	
Thrombocytopenia			0.075
Platelets < 15 × 10^4^ *μ*l	5 (19.2)	9 (7.4)	
Malignancy	3 (11.5)	3 (0.02)	0.068
Mortality rate (%)	3 (11.5)	0 (0.0)	0.006

There was no significant difference in age, gender, thrombocytopenia, and coexisting malignant disease between the groups. Seven patients with OCNF treated at our hospital survived, but 3 patients with OCNF in previous reports died, giving a mortality rate of 11.5%. This rate was significantly higher in the OCNF group (*p*=0.006, Mann–Whitney *U* test).

**Table 2:** Factors with a significant association with OCNF in univariate analysis (*χ*^2^ test).

Variable, units	OCNF (*n* = 26)	Non-OCNF (*n* = 121)	Total	*p* value
CRP, mg/dl				
≧15	22	11	33	≤0.001
<15	4	110	114	

WBC, per *μ*l				
≧15000	15	9	24	≤0.001
<15000	11	112	123	

Hb				
<11.0	7	10	17	0.052
≧11.0	19	111	130	

Cr, mg/dl				
≧1.4	10	5	15	≤0.001
<1.4	16	116	132	

GPT, IU/I				
≧100	1	1	2	0.323
<100	25	120	145	

GOT, IU/I				
≧100	1	0	1	0.177
<100	25	121	146	

Glu, mg/dl				
≧200	6	8	14	0.019
<200	20	113	133	

Alb, mg/dl				
<3.0	8	4	12	≤0.001
≧3.0	18	117	135	

Body temperature, °C				
≧38.0	4	14	18	0.590
<38.0	22	107	129	

Causative tooth				
Mandible	13	65	78	0.730
Maxilla	13	56	69	

Skin flare of cheek region				
+	16	43	59	0.014
−	10	78	88	

Skin flare of cervical region				
+	20	28	48	≤0.001
−	6	93	99	

Skin flare of precordial region				
+	9	1	10	≤0.001
−	17	120	137	

CRP, WBC, Cr, Glu, Alb, and skin flare region (cheek, neck, and precordial) showed significant differences between the OCNF and non-OCNF cases (*p* < 0.05).

**Table 3:** Factors with a significant association with OCNF in multivariate analysis (logistic regression analysis).

Variable	Logistic					
	*β*	SE	*p* value	Odds ratio	95% CI	
CRP	3.530	0.916	≤0.001	34.129	5.667	205.519
WBC	2.031	0.882	0.021	7.623	1.353	42.951
Cr	3.535	1.274	0.006	34.294	2.826	416.165
Skin flare area						
Cervical region	1.897	0.913	0.038	6.665	1.113	39.927
Precordial region	2.597	1.310	0.048	13.422	1.029	175.073

CRP, WBC, Cr, and skin flare in the cervical and precordial regions were extracted as independent factors (*p* < 0.05) and evaluated as predictors of OCNF. The multivariate-adjusted odds ratios were high for CRP (34.13, 95% confidence interval (CI) 5.67–205.52) and Cr (34.30,95% CI 2.83–416.17), indicating that these variables were the most important predictive factors. *β*: regression coefficient; SE: standard error.

**Table 4:** Laboratory Risk Indicator for Necrotizing Fasciitis of the Oro-Cervical Region (LRINEC-OC) score.

Variable	*β*	Score
CRP, mg/dl		
≧15	3.5	4
Cr, mg/dl		
≧1.4	3.5	4
WBC, per *μ*l		
≧15000	2	2
Skin flare area		
Cervical region	1.9	2
Precordial region	2.6	3

The LRINEC-OC score for prediction of OCNF was designed using the regression coefficients (*β*) in logistic analysis. The maximum total score was 15. LRINEC-OC scores of ≤5, 6–8, and 9–15 were defined as low-, moderate-, and high-risk for OCNF. Maximum score = 15. *β*: regression coefficient.

## Data Availability

The data used to support the findings of this study are available from the corresponding author upon request.
